# Comprehensive connectivity of the mouse main olfactory bulb: analysis and online digital atlas

**DOI:** 10.3389/fnana.2012.00030

**Published:** 2012-08-07

**Authors:** Houri Hintiryan, Lin Gou, Brian Zingg, Seita Yamashita, Hannah M. Lyden, Monica Y. Song, Arleen K. Grewal, Xinhai Zhang, Arthur W. Toga, Hong-Wei Dong

**Affiliations:** Laboratory of Neuro Imaging, Department of Neurology, David Geffen School of Medicine, University of California, Los AngelesLos Angeles, CA, USA

**Keywords:** main olfactory bulb, piriform cortical area, lateral olfactory tract, online digital atlas, connectome, neural tract tracing

## Abstract

We introduce the first open resource for mouse olfactory connectivity data produced as part of the Mouse Connectome Project (MCP) at UCLA. The MCP aims to assemble a whole-brain connectivity atlas for the C57Bl/6J mouse using a double coinjection tracing method. Each coinjection consists of one anterograde and one retrograde tracer, which affords the advantage of simultaneously identifying efferent and afferent pathways and directly identifying reciprocal connectivity of injection sites. The systematic application of double coinjections potentially reveals interaction stations between injections and allows for the study of connectivity at the network level. To facilitate use of the data, raw images are made publicly accessible through our online interactive visualization tool, the iConnectome, where users can view and annotate the high-resolution, multi-fluorescent connectivity data (www.MouseConnectome.org). Systematic double coinjections were made into different regions of the main olfactory bulb (MOB) and data from 18 MOB cases (~72 pathways; 36 efferent/36 afferent) currently are available to view in iConnectome within their corresponding atlas level and their own bright-field cytoarchitectural background. Additional MOB injections and injections of the accessory olfactory bulb (AOB), anterior olfactory nucleus (AON), and other olfactory cortical areas gradually will be made available. Analysis of connections from different regions of the MOB revealed a novel, topographically arranged MOB projection roadmap, demonstrated disparate MOB connectivity with anterior versus posterior piriform cortical area (PIR), and exposed some novel aspects of well-established cortical olfactory projections.

## Introduction

**Figure 1:**
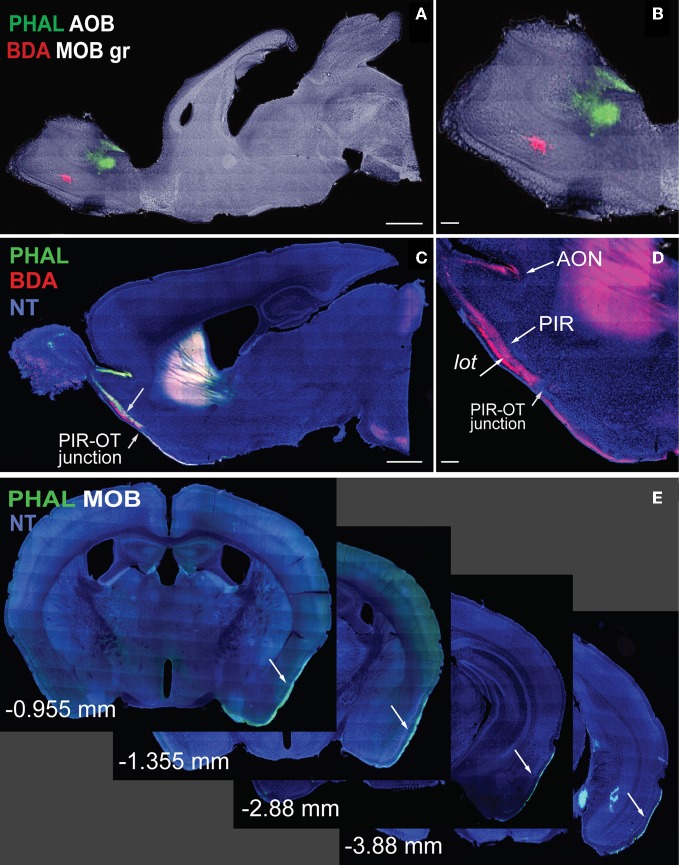
**Sagittal images of anterograde tracers *Phaseolus vulgaris-leucoagglutinin* (PHAL; green) and biotinylated dextran amine (BDA; red) injections in MOB (A, magnified in B).** Fibers originating from MOB injections travel long distances across cortical olfactory areas **(C)**. Magnified image of fibers in **(D)** shows BDA fibers in AON, PIR, and *lot*. Note the decrease in BDA axons across the PIR-OT junction **(C,D)**. Whole-brain coronal sections of PHAL fibers from MOB traveling from rostral to caudal regions of the brain (**E**; −0.955 to −3.88 mm from bregma). *Case numbers SW101215-02A **(A)**; SW101213-01A **(E)***. Scale bar, 1 mm **(A,C)**; 200 μm **(B,D)**. Abbreviations: MOB, main olfactory bulb; MOBgr, MOB granule layer; AOB, accessory olfactory bulb; PIR, piriform cortical area; OT, olfactory tubercle; AON, anterior olfactory nucleus; PHAL, *Phaseolus vulgaris-leucoagglutinin*; BDA, biotinylated dextran amine; NT, neurotrace blue.

In his pioneering work, Ramon y Cajal (1904) elegantly illustrated the cytoarchitecture of the main olfactory bulb (MOB) and its general pathways to cortical destinations using the Golgi stain. Since then, olfactory pathways have been thoroughly examined using more advanced techniques, ranging from circuit tracers to cell-type specific viral tracers (Cragg, 1961; Powell et al., 1965; Price, 1973; Scalia and Winans, 1975; Shipley and Adamek, 1984; Pro-Sistiaga et al., 2007; Yan et al., 2008; Nagayama et al., 2010; Miyamichi et al., 2011; Sosulski et al., 2011; for reviews see Haberly, 2001; Friedrich, 2011). Technological advancements also have improved presentation of neuroconnectivity data. High cost and space limitations typically allow publication of figures depicting connectivity that is of most interest, precluding presentation of the majority of the data. Unfortunately, this provides only a partial view of the whole picture. Presently, advanced imaging equipment and computer technology have revolutionized neuroanatomy such that whole-brain high-resolution images can be acquired and made publicly accessible for world-wide use (Dong, 2007; Jones et al., 2011). At the forefront of this new neuroanatomy era is the iConnecome, where systematically accumulated multi-fluorescent connectivity data can be accessed (www.MouseConnectome.org). Whole-brain coronal sections starting from rostral regions of the olfactory bulb and extending to the caudal regions of the hindbrain are available for each case. For example, olfactory cortical projections from the MOB and accessory olfactory bulbs (AOBs) extend long distances reaching the caudal ends of the entorhinal cortical area (ENT) (Figures 1A–E). Coronal sections with all of the labeling from each injection site are available in iConnectome and provide a comprehensive view of connections associated with each injected structure (Figure 1E).

### Circuit tracing approach

**Figure 2:**
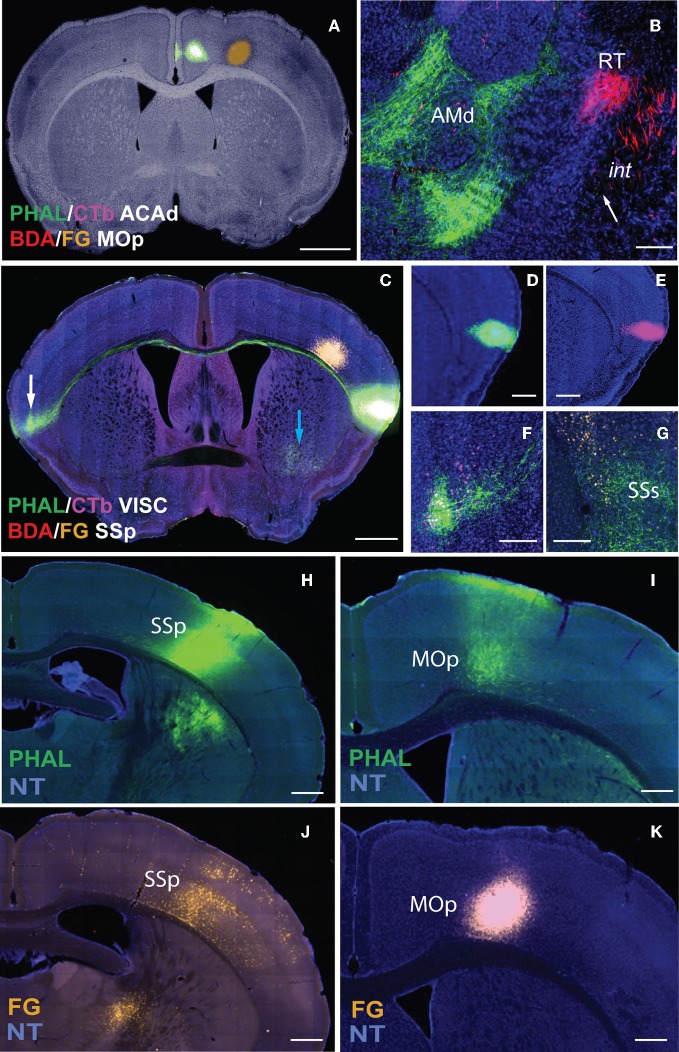
**Non-overlapping double coinjections of PHAL/CTb in ACAd and BDA in MOp on bright-field Nissl background (A) directly reveal topography in both gray (AMd and RT) and white matter (*int*; B).** White arrow indicates PHAL fibers (as dots) traveling ventral to BDA fibers in *int* **(B)**. Injections **(C–E)** also reveal long projections pathways as demonstrated by PHAL fibers from the VISC crossing the corpus callosum and terminating in the contralateral mirror VISC area also labeled with CTb neurons (**C**; white arrow magnified in **F**). Overlapping PHAL and CTb **(C,F)** labeling suggests reciprocal connectivity between the mirrored structures. Image with the VISC and SSp injections in **(C)** is exposed to reveal fine fibers (blue and white arrows) that would otherwise not be visible if adjusted for the injection. Although PHAL and CTb injections look large, their actual size is ~300 μm in diameter **(D,E)**. Possible interaction stations between the two injections sites **(C)** is indicated by PHAL fibers from VISC overlapping with FG back-labeled neurons from SSp within the supplementary somatosensory area (SSs) suggesting a VISC→SSs→SSp connectivity chain. Inter-regional connectivity is corroborated by cross validation of data **(H–K)**. PHAL injection in SSp **(H)** labels terminals in MOp **(I)**. FG injection in the same MOp site as PHAL-labeled terminals **(K)** layer-specifically back-labels neurons in SSp **(J)**, precisely in SSp PHAL injection area **(H)**. Scale bar, 1 mm **(A,C)**; 200 μm **(B,D–G)**; 500 μm **(H–K)**. *Case numbers SW110323-02A **(A,B)**; SW101014-04A **(C–G);** SW110419-03A **(H,I)**; SW110323-02A **(J,K)***. Abbreviations: VISC, visceral area; SSp, primary somatosensory area; SSs, supplemental somatosensory area; MOp, primary motor area; AMd, dorsal anteromedial thalamic nucleus; RT, reticular nucleus of thalamus; int, internal capsule.

The Mouse Connectome Project (MCP) at UCLA aims to generate a connectivity map of the mouse brain using a double coinjection tracing strategy, which was first reported for studying neuronal connectivity in the rat (Thompson and Swanson, 2010). Each of the two non-overlapping coinjections consists of one anterograde and one retrograde tracer. *Phaseolus vulgaris*-*leucoagglutinin* (PHAL: anterograde: green) is coinjected with cholera toxin subunit b (CTb: retrograde: magenta) while biotinylated dextran amine (BDA: anterograde: red) is coinjected with Fluorogold (FG: retrograde: gold) (Figure 2A). These double coinjections allow concurrent examination of input and output pathways from each injection and yield four times the amount of data collected from classic single tracer injections, reducing cost, processing time, and number of animals used. Coinjections also expose topographically distinct connectional patterns associated with the two injections within the same brain (Figure 2B), increasing the precision of a connectome map. Further, unlike MacroConnectomes that utilize *in vivo* diffusion tractography imaging to map fiber tracts (Behrens and Sporns, 2012; Cammoun et al., 2012; Van Essen et al., 2012) and MicroConnectomes (or synaptomes) (Lichtman et al., 2008; Micheva et al., 2010; Bock et al., 2011; Briggman et al., 2011) that map local circuits or synaptic connectivity at single neuron level, our approach concurrently reveals long projection pathways (Figures 2C,H–K) and inter-regional connectivity (Figures 2H–K). These inter-regional connections can be recurrent (reciprocal) connections (Figures 2C,F) and/or interaction stations (Figure 2G). Reciprocal connections between the injection sites and other structures are indicated by overlapping PHAL-labeled terminals and CTb-labeled neurons (Figures 2C,F) or by BDA terminals overlapping with FG-labeled neurons. Potential interaction stations between injection sites are demonstrated by PHAL-fiber innervation of FG-labeled neurons (Figure 2G) or BDA innervation of CTb-labeled neurons.

Inter-regional synaptic connectivity is confirmed by cross validation of the data. For example, PHAL injections in the primary somatosensory cortical area (SSp; Figure 2H) label fibers and terminal boutons in the primary motor area (MOp; Figure 2I) suggesting a synaptic connection with that region. A FG injection in the same MOp region that contains the PHAL terminals (Figure 2K) layer-specifically back-labels neurons in the SSp (Figure 2J) where the PHAL injection was made. This confirms the MOp and SSp inter-regional synaptic connection and also reveals the specific SSp layers that project to the MOp.

Sections containing the injection site are exposed to reveal fibers, which make the infusions look large (Figure 2C). However, injection sizes typically range from 300–500 μm in diameter (Figures 2D,E) and more confined injections with 200 μm diameters are made when smaller nuclei are targeted.

### The iConnectome, an online digital connectivity atlas

**Figure 3:**
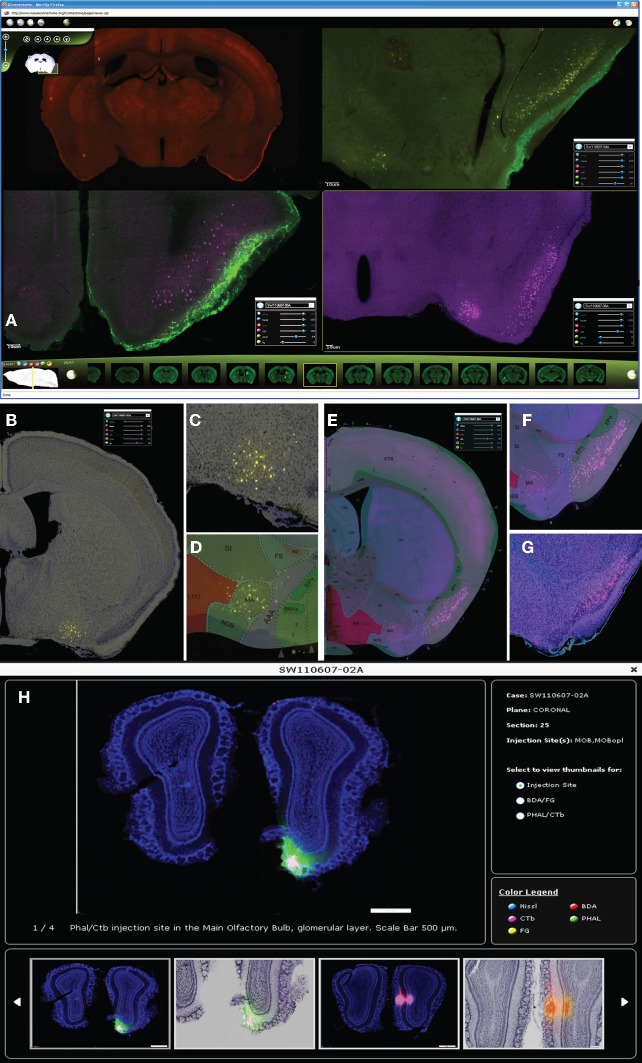
**Up to four cases can be viewed simultaneously in iConnectome for ease of comparing labeling from different cases (A).** The four fluorescent tracers can be viewed per case: BDA in the red channel (upper left); PHAL in green (upper right, lower left); CTb in magenta (lower panels), and FG in yellow (upper right). Each tracer can be viewed either on their own bright-field Nissl background **(B,C,G)** or on their corresponding ARA level **(D–F)**. Back-labeled FG neurons in MA **(B)** are magnified in **(C)** and are clearly registered onto the MA nucleus on ARA **(D)**. ARA background shows CTb back-labeled neurons in the pyramidal layer of PIR **(E)**, magnified in **(F)**. Location of neurons in layer II of PIR clearly is also seen on bright-field Nissl **(G)**. The thumbnail widget containing representative images of actual injection site size **(H)**. Thumbnails containing interesting patterns of labeling from each tracer for each case can also be found in the widget.

The iConnectome is an interactive visualization tool for our whole-brain, high-resolution connectivity data. For each case, up to four fluorescent channels, each corresponding to a tracer, can be viewed and adjusted for brightness and contrast to reveal labeling of interest (Figure 3A). PHAL is represented in the green channel (Figure 3A, upper right and lower left panels), CTb in magenta (Figure 3A, lower panels), BDA in red (Figure 3A, upper left), and FG in yellow (Figure 3A, upper right). Up to four different cases (approximately 16 pathways) can be viewed simultaneously (Figure 3A). Windows can be synchronized such that any action performed in the master viewport is mirrored in the slave window(s) allowing comparison of labeling patterns from different cases.

The purpose of this publicly accessible connectivity data is to help neuroscientists generate testable hypotheses regarding finer scale brain circuitry, brain function, behavior, and disease. Two features that ease the analysis of the connectivity data are available in iConnectome. The first is a channel that allows each section to be viewed within its own bright-field Nissl cytoarchitectural background (Figures 3B,C,G). Previously, this has not been attainable due to technical constraints and consequently adjacent sections commonly have been used as Nissl references. The bright-field background of the same section enables more precise identification of labeling in distinguishable nuclei, decreasing the margin of error in analysis. The second feature is a sixth channel that represents the section's corresponding Allen Reference Atlas (ARA; Dong, 2007) level, which also aids in the annotation of the data (Figures 3D–F).

For each case, a thumbnail widget containing representative images also is available. The widget can be accessed by clicking on the magnifying glass icon next to each case number (Figure 3H). This feature provides an overview of the labeling for the case, but more importantly, displays the actual injection site size. As aforementioned, injection sites are over exposed to reveal finer labeling present in the section (Figure 2C), which enlarges the infusion area and misrepresents the injection size.

Currently, the iConnectome features 63 cases, 18 of which contain MOB injections that trace approximately 35 efferent and 20 afferent pathways across the entire brain. Eventually, injections made into the AOB, anterior olfactory nucleus (AON), and other cortical olfactory areas also will be available.

## Results

Analysis of injections made into the mitral cell (MOBmi) and granule (MOBgr) layers of the MOB and into the piriform cortical area (PIR) revealed novel topographic MOB projections to and within the *lot*, demonstrated different MOB connections with the anterior versus posterior PIR, and exposed novel characteristics of well-established cortical olfactory projections.

### Projection roadmap of the MOB: route preferences of lateral versus medial MOB mitral cells

**Figure 4:**
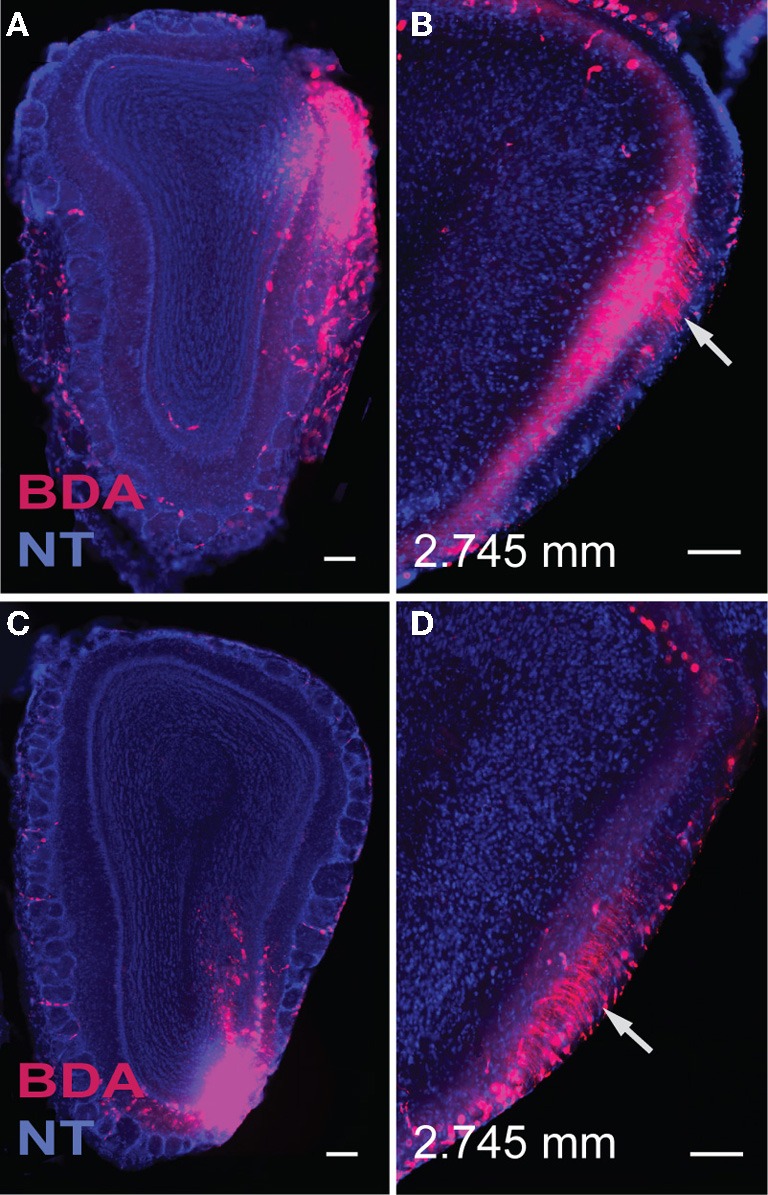
**Injections in the dorsal (A) and ventral (C) *lateral* MOB result in axons traveling directly toward the *lot* located on the same side.** Within the *lot*, fibers from the dorsal *lateral* MOBmi travel predominantly in dorsal parts of the *lot* **(B)**, while those from ventral *lateral* regions remain primarily in ventral *lot* **(D)** as they travel caudally toward their olfactory cortical destinations. Sections containing the *lot* are at the same level, approximately 2.745 mm anterior from bregma **(B,D)**. Scale bar, 200 μm. *Case numbers SW110608-05A **(A,B)**, SW110607-03A **(C,D)***.

**Figure 5:**
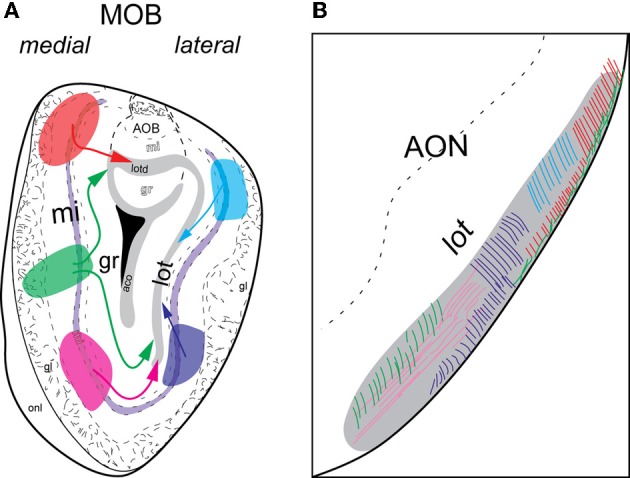
**Schematic of *lateral* and *medial* MOB projections to *lot*.** Colors of injections in **(A)** and *lot* fibers in **(B)** are linked. Axons from dorsal (blue) or ventral (purple) *lateral* MOB travel directly toward the *lot* on the same side **(A)** and remain approximately in the dorsal and ventral *lot*, respectively **(B)**. Fibers from dorsal *medial* MOB (orange) travel through the *lotd*, while those from ventral *medial* MOB (pink) take a ventrolateral route. Fibers originating from the middle *medial* MOB (green) travel either through the *lotd* or ventrolaterally toward *lot*. Within the *lot*, dorsal *medial* MOB fibers stay restricted roughly within the lateral or dorsolateral region. Axons from ventral *medial* MOB remain approximately in ventromedial region and those from middle *medial* MOB travel through either dorsolateral or ventromedial *lot*. Abbreviations: AOB, accessory olfactory bulb; AON, anterior olfactory nucleus; lot, lateral olfactory tract; lotd, dorsal limb of lot.

**Figure 6:**
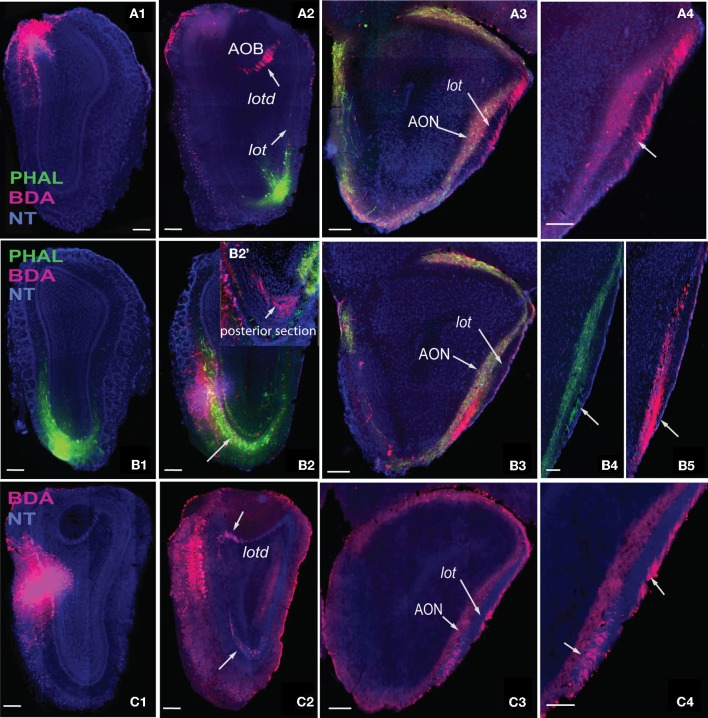
**Axons from the dorsal *medial* MOB (A1) travel through *lotd* (A2) to *lot* and travel along the dorsolateral edge within the *lot* (A3,A4).** Ventral *medial* MOB axons **(B1,B2)** travel ventrolaterally toward *lot* **(B2,B2')** and within the ventromedial parts of *lot* **(B3–B5)**. **B2'** is one section posterior to section in **B2**. Axons from middle *medial* MOB **(C1)** cross over either through the *lotd* or ventrolaterally toward the *lot* **(C2)** and travel either through its dorsolateral or ventromedial parts **(C3,C4)**. Scale bar, 200 μm. *Case numbers SW101213-01A **(A)**, SW101215-01A **(B)**, SW100302-01A **(C)***.

Double coinjections were made into the (1) dorsal, (2) middle, or (3) ventral MOBmi along the dorsal-ventral axis and into *medial* or *lateral* regions along the medial-lateral axis (Figures 4A,C, 5A, 6A1,A2,B1,B2,C1).

Regardless of their origin along the dorsal-ventral axis, axons from the *lateral* MOB course through the granule layer headed toward the *lot*, the main route from the olfactory bulb to olfactory cortical areas (Gloor, 1997) (Figures 4A,C, 5A). Within the *lot*, axons from the dorsal *lateral* region travel roughly in dorsal intermediate parts of the tract, while axons from the ventral *lateral* MOBmi travel roughly in the ventral intermediate portion (Figures 4B,D, 5B) before arborizing in the AON and PIR.

Axons from the *medial* MOB take different routes to join the *lot* depending on their origin along the dorsal-ventral axis. Those from dorsal *medial* MOBmi (Figure 6A1) travel through the dorsal limb of the *lot* (*lotd*; Figure 6A2) and through a distinct dorsolateral and lateral region of the *lot* (Figures 6A3,A4). From the ventral *medial* MOBmi, axons travel ventrolaterally across the MOB toward the *lot* (Figures 6B1,B2,B2') and extend caudally roughly through the ventromedial *lot* (Figures 6B3–B5). Axons from the middle *medial* MOBmi (Figure 6C1) travel either dorsally via the *lotd* or go ventrolaterally toward the *lot* (Figure 6C2). Axons from the *lotd* occupy the dorsolateral or lateral edge of the *lot*, while axons traveling ventrolaterally occupy more ventromedial parts (Figures 6C3,C4; see Figure 5 for schematic).

### Differential connectivity patterns of the anterior versus posterior PIR

**Figure 7:**
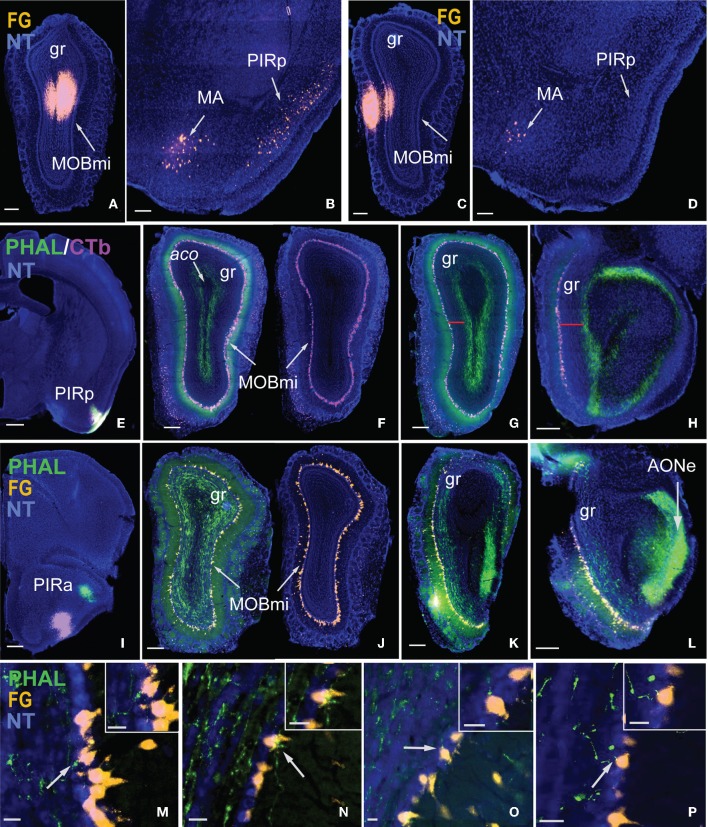
**Stratification of MOBgr and differential connectivity patterns of PIRa and PIRp with MOB.** Injections in deep MOBgr **(A)** retrogradely label neurons in both MA and PIRp **(B)**, while injections in MOBmi **(C)** label only MA neurons and not PIRp **(D)**. PHAL injections into PIRp **(E)** label axons only in deep MOBgr **(F)**, a pattern that is preserved in posterior sections **(G,H)**. Note the distance from CTb labeled mitral cells and PHAL labeled fibers in **G,H** indicated by a red bar. PIRa projects to MOBmi and more superficial MOBgr **(I,J)**, which is also apparent in posterior MOBgr **(K,L)**. Note the distance between FG labeled mitral cells and PHAL labeled fibers. **(M–P)** are magnified images from **(J)** showing PHAL-labeled terminal boutons from PIRa contacting FG-labeled mitral cells. White arrows indicate magnified cells in top right corner of image. Scale bar 200 μm **(A–D,F–L)**; 500 μm **(E)**; 20 μm **(M–P)**. *Case numbers SW101215-05A **(A,B)**, SW110607-05A **(C,D)**; SW110403-01A **(E–H)**, SW110616-04A **(I–L)***. Abbreviations: MA, magnocellular nucleus; PIRp, posterior piriform area; PIRa, anterior piriform area; aco, anterior commissure olfactory limb; MOBmi, MOB mitral cell layer; gr, granule layer of MOB; AONe, external anterior olfactory nucleus.

**Figure 8:**
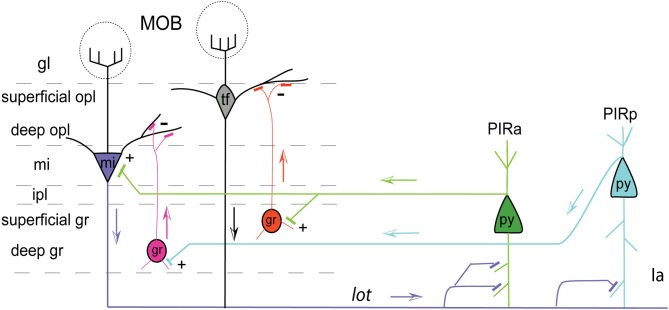
**Pyramidal cells of PIRa (green) send excitatory projections (indicated by +) to MOB mitral cells (mi; purple).** PIRa (green) also directly projects to superficial MOB granule cells (gr; orange), which in turn inhibit (indicated by –) tufted cells (tf) in the superficial outer plexiform layer (opl). Pyramidal cells of PIRp (teal) project only to deep granule cells (gr; pink), which inhibit mi cells. Axons from MOB mi cells (mi; purple), through the *lot*, project more densely to PIRa compared to PIRp indicated by more synaptic connections. Apical dendrites of PIRa (py; green) and PIRp (py; teal) pyramidal neurons form connections with MOB mi projecting axons traveling within layer Ia. Arrows indicate flow of direction. Abbreviations: gl, glomerular layer; opl, outer plexiform layer; mi, mitral cell layer; ipl, inner plexiform layer; gr, granule layer; Ia, molecular layer 1a.

Injections made into the MOBgr revealed unique connections of the MOB with the anterior and posterior PIR. FG injections encompassed within the dorsal deep MOBgr (Figure 7A) result in back-labeled neurons both in the posterior PIR (PIRp) and magnocellular nucleus (MA; Figure 7B). FG injections in the MOBmi that encroach slightly onto the superficial granular layer (Figure 7C) label neurons only in the MA, not the PIRp (Figure 7D). This suggests that the MA projects to both MOBgr and MOBmi, but that the PIRp projects only to the deep MOBgr. Corroborating this connection, PHAL/CTb injections in the PIRp (Figure 7E) result in labeled terminals solely in the deep MOBgr (Figure 7F), a pattern that is preserved in posterior MOBgr regions (Figures 7G,H). PIRp CTb injections confirm that cells in the entire MOBmi project back to the PIRp (Figures 7F–H). Together, the data suggest a connection chain from the MOBmi→PIRp→deep MOBgr. PHAL injections in the anterior PIR (PIRa) specifically innervate the superficial MOBgr layers and the MOBmi (Figures 7I,J,M–P). This pattern also is preserved in more posterior regions of the MOB (Figures 7K,L) and suggests a neural chain from MOBmi→PIRa→MOBmi/superficial MOBgr. Combined, these results demonstrate that (a) the MOBgr can be stratified into superficial and deep layers and (b) the PIRa and PIRp show differential connectivity patterns to the MOB, namely that the PIRa projects to the superficial MOBgr and MOBmi, while PIRp projects to deep MOBgr and avoids the MOBmi (Figure 8). This distinct connectivity of the PIRa and PIRp possibly has important implications for their roles in MOB activation (see “Discussion”).

**Figure 9:**
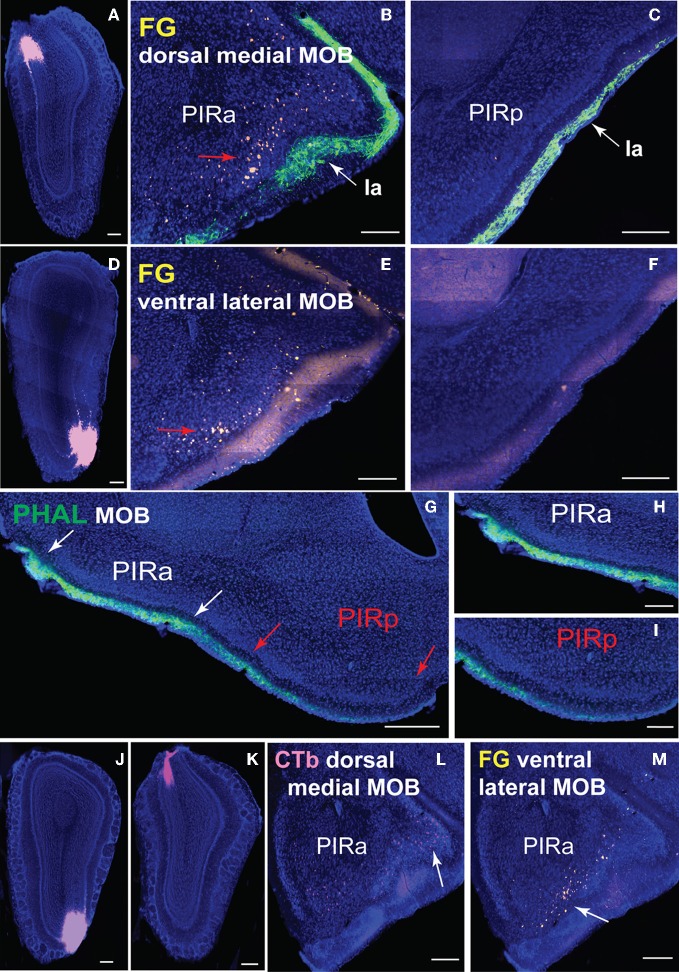
**Connectional differences between PIRa and PIRp.** FG injections in dorsal *medial* **(A)** and ventral *lateral* **(D)** MOBmi back-label neurons in PIRa **(B,E)**, but not in PIRp **(C,F)**. Layer Ia of PIR, labeled with PHAL (see Figure 6A2 for PHAL injection), appears to get thinner in width from PIRa to PIRp (**B,C,E–I**). Input to PIRa and PIRp is also different as MOB projects more densely to PIRa compared to PIRp **(G–I)**. PIRa between white arrows **(G)** is magnified in **(H)**, while PIRp in between red arrows **(G)** is magnified in (**I**; see Figures 1A,B for PHAL injection). Topographic arrangement of neurons in PIRa (red arrows in **B,E**) are more clearly observed when FG and CTb are double injected in dorsal versus ventral MOBmi, respectively **(J,K)**. Dorsal MOBmi projecting CTb neurons occupy more dorsal regions of PIRa **(L)**, while ventral projecting neurons are in more ventral parts of PIRa **(M)**. Scale bar, 200 μm; 500 μm **(G)**. *Case numbers SW101213-01A **(A–C)**, SW101215-03A **(D–F)**, SW101215-02A **(G–I)**, SW110607-03A **(J–M)***.

Differential PIRa and PIRp connectivity is substantiated by FG injections in the *medial* and *lateral* MOBmi that back-label neurons only in the PIRa and not PIRp (Figures 9A–F). This pattern holds true regardless of the dorsal-ventral position of the MOB injections (data not shown). Our data also suggests that PIRa receives more inputs from MOBmi than the PIRp. From a sagittal view, MOB fiber ramifications decrease as they progress from PIRa to PIRp (Figures 9G–I).

A rough topography within PIRa also exists where more dorsal neurons project to dorsal MOBmi and more ventral PIRa neurons project to ventral MOBmi (Figures 9B,E). This coarse organization is observed more clearly when CTb and FG are double injected in the dorsal and ventral MOBmi, respectively (Figures 9J,K). CTb neurons roughly cluster in more dorsal parts of the PIRa, while FG neurons occupy more ventral regions (Figures 9L,M).

### MOB cortical projections

**Figure 10:**
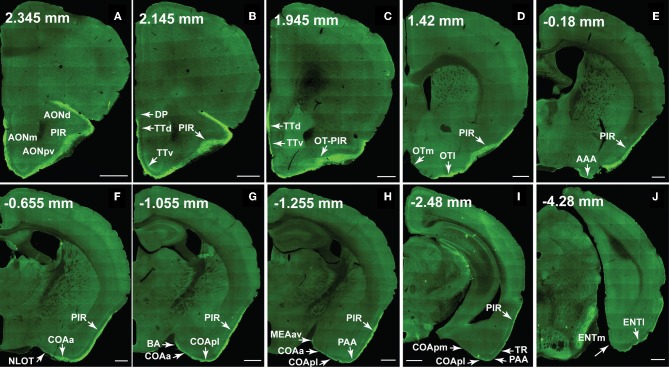
**The ventral *lateral* MOB projects to olfactory cortical structures from the AON to the ENT (2.345 to –4.28 mm from bregma).** Axons ramify in AONd, AONm, AONpv **(A)**, TTd, TTv **(B,C)**, PIR **(A–I)**, OTl **(D)**, AAA **(E)**, NLOT **(F)**, COAa **(F–H)**, COApl **(G–I)**, BA **(G)**, MEAav **(H)**, PAA **(H,I)**, TR **(I)**, and ENTl **(J)** without any spatial topography. The DP, OTm, COApm, and ENTm do not receive inputs from the ventral *lateral* MOBmi (**B,D,I,J**). The PIR-OT junction contains dense labeling **(C)**, where the fibers appear to wrap around layer II pyramidal neurons in OTl (Figures 12H,I). Axon numbers appear to decrease as they travel medially **(B–J)** and caudally **(A–J)**. Differences between projection patterns from the ventral *lateral* and dorsal *medial* MOBmi were not detected (see Figure 11). *Case number SW101213-01A*. Scale bar, 500 μm. Abbreviations: AONd, dorsal anterior olfactory nucleus; AONm, medial AON; AONpv, posterior ventral AON; TTd, dorsal taenia tecta; TTv, ventral TT; PIR, piriform area; OTl, lateral olfactory tubercle; OTm, medial OT; AAA, anterior amygdalar area; NLOT, nucleus of lateral olfactory tract; COAa, anterior cortical amygdalar area; COApm, posterior medial COA; BA, bed nucleus of accessory olfactory tract; MEAav, anterior ventral medial amygdalar nucleus; PAA, piriform-amygdalar area; TR, postpiriform transition area; ENTl, lateral entorhinal area; ENTm, medial ENT.

**Figure 11:**
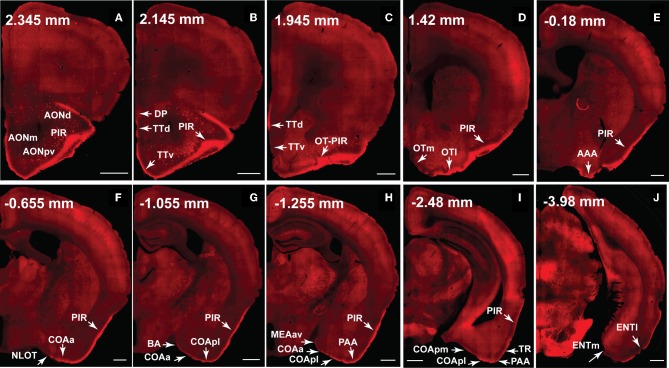
**The dorsal *medial* MOB projects to olfactory cortical structures from the AON to the ENT (2.345 to –3.98 mm from bregma).** Axons ramify in AONd, AONm, AONpv **(A)**, TTd, TTv **(B,C)**, PIR **(A–I)**, OTl **(D)**, AAA **(E)**, NLOT **(F)**, COAa **(F–H)**, COApl **(G–I)**, BA **(G)**, MEAav **(H)**, PAA **(H,I)**, TR **(I)**, and ENTl **(J)** without any spatial topography. The DP, OTm, COApm, and ENTm do not receive inputs from the dorsal *medial* MOBmi **(B,D,I,J)**. The PIR-OT junction contains dense labeling **(C)**, where the fibers appear to wrap around layer II pyramidal neurons in OTl (Figures 12H,I). Axon numbers appear to decrease as they travel medially **(B–J)** and caudally **(A–J)**. Differences between projection patterns from the dorsal *medial* and ventral *lateral* MOBmi were not detected (see Figure 10). *Case number SW101213-01A*. Scale bar, 500 μm. Abbreviations: AONd, dorsal anterior olfactory nucleus; AONm, medial AON; AONpv, posterior ventral AON; TTd, dorsal taenia tecta; TTv, ventral TT; PIR, piriform area; OTl, lateral olfactory tubercle; OTm, medial OT; AAA, anterior amygdalar area; NLOT, nucleus of lateral olfactory tract; COAa, anterior cortical amygdalar area; COApm, posterior medial COA; BA, bed nucleus of accessory olfactory tract; MEAav, anterior ventral medial amygdalar nucleus; PAA, piriform-amygdalar area; TR, postpiriform transition area; ENTl, lateral entorhinal area; ENTm, medial ENT.

**Figure 12:**
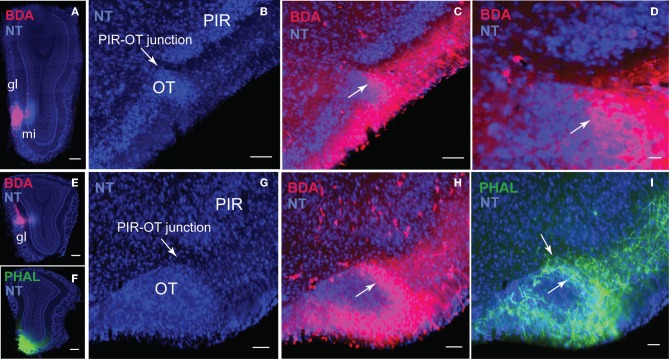
**Dense projections from different regions of the MOB including the MOBgl and MOBmi (A,E,F) to the junction between the PIR and OT (B–D,G–I). (C)** is magnified in **(D)** to demonstrate how axons wrap around pyramidal OT neurons **(H,I)**. Scale bar, 100 μm; 20 μm **(D)**. *Case numbers SW101213-04A **(A–D)**; SW110607-02A **(E–I)***. Abbreviations: gl, glomerular layer of MOB; mi, mitral cell layer of MOB; PIR-OT, piriform-olfactory tubercle junction.

Double coinjections were made into the dorsal *medial* (BDA; Figure 6A1) and ventral *lateral* (PHAL; Figure 6A2) MOBmi and fibers from the rostral to caudal regions of the brain were examined. Regardless of their origin, all axons extensively arborize along the molecular Ia sublayer of the olfactory cortex without any spatial topographic specificity. From the *lot*, fibers from dorsal *medial* and ventral *lateral* MOBmi first arborize in the AON, extending across its external, dorsal, lateral, and posterior ventral divisions (Figures 10A, 11A). Caudally, fibers continue into the Ia layer of the PIR, dorsal/ventral taenia tecta (TTd, TTv), but do not project as far mediodorsal as the dorsal peduncular area (DP; Figures 10B, 11B). Axons densely ramify at the juncture between the PIR and the olfactory tubercle (OT; Figures 10C, 11C). Several cases substantiate this PIR-OT junction labeling where it appears that axons from different parts of the MOB, including the MOBmi and glomerular layer (MOBgl; Figures 12A,E,F), extend into layer II and wrap around pyramidal OT neurons (Figures 12B–D,G–I).

Fibers continue caudally and terminate in the lateral OT, avoiding its medial portion across the rostral-caudal extent of the structure (Figures 10D, 11D). PHAL and BDA MOBmi axons also terminate in the anterior amygdalar area (AAA; Figures 10E, 11E), nucleus of the olfactory tract (NLOT; (Figures 10F, 11F), bed nucleus of accessory olfactory tract (BA; Figures 10G, 11G), anterior cortical amygdalar area (COAa; Figures 10F–H, 11F–H), posterior lateral COA (COApl; Figures 10G–I, 11G–I), anterior ventral part of the medial amygdalar nucleus (MEAav; Figures 10H, 11H), piriform-amygdalar area (PAA; Figures 10H,I, 11H,I), and postpiriform transition area (TR; Figures 10I, 11I). Terminal boutons are not present in the posterior medial COA (COApm; Figures 10I, 11I). Similarly, arborizations are observed in the Ia layer of the lateral entorhinal cortical area (ENTl), but not the medial ENT (ENTm; Figures 10J, 11J).

The number of axons progressively decreases not only as they project medially, but also caudally. For example, massive axons in the AON and PIR dramatically decrease in number after the PIR-OT juncture (Figures 10C,D, 11C,D), an observation that is more evident in sagittal sections (Figure 1C).

## Discussion

### MOB projection roadmap

An important question in the olfactory field is whether the exquisite spatial arrangement of olfactory sensory neuron (OSN) inputs to olfactory bulb glomeruli is preserved in connections to higher brain regions (Isaacson, 2010). In rodents, each OSN class expresses a unique odorant receptor set and has projections to two glomeruli that are located in symmetrical positions of the olfactory bulb: one on the medial and the other on the lateral side (Ressler et al., 1994; Vassar et al., 1994; Mombaerts et al., 1996). As a consequence of this topography, each olfactory receptor, and the odors that activate it, is represented in the olfactory bulb as a pattern of activated glomeruli. Imaging experiments have validated that different odorants elicit distinct patterns of glomerular activity (Rubin and Katz, 1999). Each of these symmetrically located glomeruli receives input from mitral cells located in the same symmetric positions and evidence suggests existence of organized projections from glomeruli to AON (Franks et al., 2011). Predicated on this organization, we purported that symmetrically located mitral cells in the *medial* and *lateral* regions of the MOB would have similar projection patterns, while those located in different positions along the dorsal-ventral axis would differ. Our projection roadmap of the MOB supports this claim. Once within the *lot*, fibers from the dorsal *medial* and *lateral* MOB travel roughly within the dorsal region of the tract, while those in the ventral *medial* and *lateral* travel approximately in its ventral portion (Figures 4, 5, and 6). Therefore, within the *lot*, positions of axons from symmetrical *medial* and *lateral* MOB locations are topographically similar, while positions of *lot* axons from dorsal and ventral MOB locations differ. Support for this dorsal/ventral *lot* stratification is provided by differential expression of molecular markers within the fiber tract (Inaki et al., 2004). Our data did not show spatial arrangements from *lot* to layer Ia of olfactory cortical areas, which is consistent with the majority of reports in the literature (Ghosh et al., 2011; Kang et al., 2011a; Sosulski et al., 2011), although some evidence suggests that the OT preferentially receives more inputs from the dorsal MOB exists (Haberly and Price, 1977).

The olfactory pathways have been studied extensively over the last several decades (for reviews see Gloor, 1997; Haberly, 2001). Consistent with the literature, our data show that axons arising from different parts of the MOB generate axonal projections to other olfactory cortical areas including the AON, TTd, TTv, PIR, MEA, COA, and ENT as well as to the OT, which forms part of the ventral striatum. Generally, MOB projections stay restricted to the lateral division of structures i.e., the lateral OT, COApl, and ENTl, avoiding the medial OT, COApm, and ENTm. Unlike most cortical projecting MOB axons that remain restricted in the molecular Ia layer, MOB axons from all regions generate terminals in the junction between the PIR and OT while the axons surround the OT layer II pyramidal neurons. This suggests that unlike pyramidal neurons in other olfactory cortical areas that receive MOB inputs via the distal portion of their apical dendrites, MOB axons potentially form direct connections onto somas and/or dendrites of OT neurons.

Finally, the amount of MOB axons progressively decreases as they project medially and caudally. The massive number of axons in the AON and PIR dramatically decrease as they reach the OT. They decrease even further in more caudal structures like COA and ENT. This is observed on both coronal and sagittal sections and across several cases. There are two possible explanations for this phenomenon: (1) not all axons travel the entire course. Some axons may terminate specifically in the PIR or COA, while others extend all the way to ENTl or (2) the axons do extend through the entire course, but generate less collaterals and arborizations toward the caudal end of the brain. This question can be investigated by genetically labeling individual neurons in the MOB and examining the morphological details of their long axonal projections.

### MOB projections to the PIR and other cortical areas

There is contradictory information regarding MOB inputs to the PIR. Earlier reports assert that afferents from olfactory bulb synapse primarily onto neurons in the PIRa and only send lighter, more distributed inputs to PIRp (Haberly and Price, 1978; Shipley and Adamek, 1984; Haberly, 2001). In contrast, genetic tracing methods have reported that axons from individual glomeruli project diffusely to the entire PIR without apparent spatial preference (Sosulski et al., 2011). A possible explanation for these contradictory observations is that flattened hemi-brain preparations were used for some studies (Sosulski et al., 2011), while coronal sections were used in others. While a flattened hemi-brain preparation facilitates the observation of the axonal arborizations on the tangential plane, coronal sections reveal the entire scope of axonal ramifications.

Based on our data from coronal sections, distinct projection patterns from the MOBmi to PIRa versus PIRp are not evident. However, progressing from PIRa to PIRp, layer Ia appears to get thinner in width while the Ib thickens (Figures 9B,C,E–G). If this truly is the case and since Ia is the specific layer in which ramifications from MOB projections occur, this suggests that PIRa receives more inputs from the MOB compared to PIRp. Although differences in axon numbers are not discernible from the coronal plane, it is clear from sagittal sections that MOB fibers decrease as they travel from PIRa to PIRp (Figures 9G–I). Such patterns (i.e., number of MOB axons and layer width) potentially have implications for the connections formed between the MOB and these structures. It is possible that MOB axons form synaptic connections along the entire length of dendrites of AON and PIRa pyramidal neurons given their thicker and more densely populated Ia layer. In contrast, in the PIRp, COA, and ENT, potentially only pyramidal neurons with long apical dendrites that reach the outermost regions of a relatively thinner Ia can receive input from the far fewer MOB axons traveling through these areas. In fact, it is reported that the ratio of associative layer Ib to afferent Ia inputs is higher in more posterior regions of the PIR (Haberly, 1998).

The anatomical similarities between the AON and PIRa (Figures 1C,D) may be important for understanding how olfactory information is processed. The two structures can serve as the first association relay station between the MOB and caudal olfactory cortex. The AON is critical for synchronizing and integrating olfactory information from both sides of the bulb (Yan et al., 2008), while PIRa neurons encode sensory features of olfactory cues and relay this information rostrocaudally along the olfactory cortex (Haberly, 2001; Calu et al., 2007; Roesch et al., 2007). This information could be processed further in the second association area, the PIRp, which integrates olfactory information with inputs from the amygdala, entorhinal, medial prefrontal, orbitofrontal, and insular cortices that process multimodal and associative information (Johnson et al., 2000; Haberly, 2001). Functional data also suggests that PIRp neurons are involved in higher order processing compared to the PIRa. For example, the PIRp processes the predictive value of olfactory cues, implicating its involvement in associative olfactory learning (Calu et al., 2007; Roesch et al., 2007). This also is demonstrated in humans where PIRp, rather than the PIRa, generates olfactory predictive codes to augment olfactory perception based on previous experience (Zelano et al., 2011), which is important for orienting selective attention toward an odor of interest and away from an odor of no interest upon subsequent exposures. Since the PIRp projects directly to the deep MOBgr it may then indirectly inhibit further outputs from mitral cells to olfactory cortices (discussed below).

### Distinct PIR projections to MOB

It is reported that the PIR receives direct mitral cell input representing glomeruli from different regions of the olfactory bulb with no apparent spatial organization (Miyamichi et al., 2011). Our data support this result since CTb injected into the PIR non-preferentially back-labels mitral cells throughout the entire bulb (Figure 7F). Although MOB efferents suggest no topographic connectivity with the PIR, distinct connectional patterns are exposed when examining projections from the PIR to the MOBmi. Inputs to MOBmi show that while the PIRa projects to the MOBmi and to the superficial MOBgr, PIRp projects only to the deep MOBgr. In the basic organization of the olfactory system, GABAergic granule cells via dendrodentritic synapses (Gloor, 1997) act to inhibit the glutamatergic mitral or tufted cells (tfs), whose activation is the principal means of mediating output control of the olfactory bulb. This MOBgr stratification (deep versus superficial layers) is supported by data demonstrating that granule cells segregate into at least three different subpopulations based on morphological and molecular criteria. Deep granule cells have their dendritic arbors restricted to the deep outer plexiform layer (opl) where they are believed to synapse predominately with mitral cell secondary dendrites. In contrast, superficial granule cells arborize in the superficial opl where they synapse with tf dendrites (for review see Shepherd et al., 2007). Together with our data, this suggests that the PIRa possibly provides direct excitatory inputs to the MOBmi (MOBmi→PIRa→MOBmi), while its inputs to the superficial MOBgr cells provide an indirect inhibitory feedback control of MOBtf cells (MOBmi/tf→PIRa→superficial MOBgr→MOBtf). On the other hand, PIRp could indirectly inhibit the MOBmi cells via its projections to deep granule cells (MOBmi→PIRp→deep MOBgr→MOBmi; see Figure 8 for schematic).

Our data also revealed a coarse topography within PIRa where more dorsal PIRa neurons tend to project to dorsal MOBmi, while more ventral PIRa neurons project to more ventral MOBmi. This is observed more clearly in a case where CTb and FG were double injected in the dorsal and ventral MOBmi, respectively, in the same animal. CTb neurons clustered in more dorsal parts of the PIRa, while FG roughly occupied more ventral regions. It should be noted that based on the data at hand, a clear boundary between the PIRa and PIRp or dorsal and ventral PIRa cannot be defined. Setting hard boundaries would require a more comprehensive examination of PIR connectivity, which will be performed in the future.

### MOB projections to classic accessory olfactory structures

The rodent olfactory system consists of two parallel chemosensory systems: the vomeronasal (VNO) or “accessory olfactory” and main olfactory systems. Classically, the VNO is thought to process non-volatile pheromones that lead to stereotyped endocrinological responses like aggression, acceleration or suppression of estrus, and pregnancy block (for review see Halpern, 1987). Primary projections of the AOB critical for pheromone processing include the BA, MEAa, MEApd, MEApv, COApm, and bed nuclei of stria terminalis (BST; principle nucleus) (Halpern, 1987; Simerly, 1990; Gloor, 1997). The main olfactory system primarily processes more complex, volatile chemosensory cues that are modifiable through experience (Halpern, 1987) and involve MOB projections to the AON, OT, and ENT (Gloor, 1997; Haberly, 2001). This strict segregation of the VNO and MOB systems has been challenged as research has shown pheromone activation of the MOB and MOB projections to more classic accessory structures like BA and MEAa, MEApd (Martínez-García et al., 1991; Pro-Sistiaga et al., 2007; Kang et al., 2011a,b). Our data also show MOBmi projections to BA and MEAav lending support for the possibility that the classic views of the main and accessory olfactory systems require modification. Fortifying this view is data showing prototypal olfactory recipients such as NLOT, COAa, and PAA also receive significant inputs from the AOB (Pro-Sistiaga et al., 2007). Thus, the MEAav, BA, NLOT, COAa, and PAA may form a third “integrated” category of the olfactory system, which receives and processes converged information from both AOB and MOB. Evidence for complementary roles for the main and AOBs substantiates this possibility of integrated VNO and MOB systems (Martínez-García et al., 2009).

### Concluding comments

We provide the first open resource for olfactory pathways, which gradually will be expanded to include structures from the entire olfactory system. This connectivity database will generate testable hypothesis for studying the olfactory system and its interactions with structures such as the amygdala, hippocampus, medial prefrontal cortical area, and thalamus.

Our data showed topographically organized projection patterns of the *medial* MOBmi. Similar to reports in the literature, there was no clear spatially topographic projection from the MOB to PIR. However, we did find that axons from the MOB project more densely to PIRa than to PIRp. Further, we showed a clear topographic projection from the PIRa and PIRp to the MOB. This evidence suggests that the PIRa possibly provides direct excitatory inputs to the MOBmi, while its inputs to the superficial MOBgr cells provide an indirect inhibitory feedback control of MOBtf cells. It also suggests that the PIRp inhibits MOBmi via it projections to deep MOBgr cells. This hypothesis can be further tested and may shed light on the mechanisms underlying olfactory related behaviors.

## Materials and methods

### Subjects and husbandry

Data from 17 eight-week old male C57Bl/6J mice from Jackson Laboratories were used. They were housed in pairs in a temperature (21–22°C), humidity (51%), and light controlled (12 h light:12 h dark cycle with lights on at 6:00 am and off at 6:00 pm) vivarium. Mice were allowed 1 week to adapt to their living conditions before surgery. Experiments were conducted according to the standards set by the National Institutes of Health Guide for the Care and Use of Laboratory Animals and the institutional guidelines of the University of California, Los Angeles.

### Surgeries

To comprehensively examine MOB connectivity patterns, double coinjections were made into the (1) dorsal, (2) middle, or (3) ventral MOBmi along the dorsal-ventral axis and into *medial* and *lateral* regions along the medial-lateral axis. Injections also were made into the MOBgr and PIR (coordinates for each injection are available at www.MouseConnectome.org. Mice were initially anesthetized in an induction chamber primed with isoflurane and subsequently mounted to the stereotaxic apparatus where they were maintained under anesthetic state [2.5 gas mixture with oxygen (0.5 L/min) and nitrogen (1 L/min)]. PHAL/CTb and BDA/FG infusions were delivered iontophoretically using glass micropipettes (O.D. ~15–20 μm). A positive 5 μAmp, 7 s alternating current was delivered for 10 min. The analgesic buprenorphine was administered the day of and day following surgeries (0.05 mg/kg). Animals were sacrificed with an overdose injection of pentobarbital (6 mg/kg) 7 days following surgeries.

### Tracers

PHAL (2.5%; Vector Laboratories) was coinjected with CTb (647 conjugate, 0.25%; Invitrogen), while BDA (FluoroRuby, 5%; Invitrogen) and FG (1%; Fluorochrome, LLC) were injected in combination.

### Tissue preparation

Each animal was transcardially perfused with approximately 50 ml of 0.9% NaCl followed by 50 ml of 4% paraformaldehyde solution (PFA; pH 9.5) following an overdose injection of sodium pentobarbital. Brains were post-fixed in 4% PFA for 24–48 h at 4°C after which they were embedded in 3% Type I-B agarose and sectioned into four series of coronal sections at 50 μm thickness.

### Immunofluorescence staining

One of four series was stained for PHAL using the free-floating method. Briefly, sections were transferred to a blocking solution (normal donkey serum and Triton X) for one hour. Following three 5 min rinses, sections were incubated with 1:1000 concentration of rabbit anti-PHAL antibody for 48–72 h at 4°C along with donkey serum and Triton. Sections were rinsed three times in KPBS and then soaked for 3 h in the secondary antibody solution (1:500 concentration of anti-rabbit IgG conjugated with Alexa Fluor^®^ 488). Sections were counterstained with the fluorescent Nissl stain NeuroTrace^®^ 435/455 (NT; 1:1000), mounted, and coverslipped using 65% glycerol.

### Imaging and post-acquisition processing

Sections were imaged using an Olympus VS110 virtual slide scanner. Each image was flipped to the correct left-right orientation, matched to the nearest ARA (Dong, 2007) atlas level, and converted to tiff format prior to being registered (discussed below). Following registration and registration refinement, the fluorescent Nissl was converted to bright-field and each image for each channel (PHAL, CTb, BDA, FG, and NT) manually was adjusted for brightness and contrast to maximize labeling visibility and quality in iConnectome. Following final modifications (i.e., skewness, angles, preparation of images for thumbnail widget) and pyramidal tiff conversions, images were published to iConnectome.

Distracting artifacts from BDA that may have been mistaken for labeling were removed from the images. Labeling (axons, boutons, neurons) for the images was not manipulated and all original raw images are available on iConnectome.

### Semi-automated image registration

To ease analysis of the connectivity data, each section was registered onto its corresponding ARA atlas level (Figures 3D–F). A combination of automatic and manual registration steps was used to resample, align, and co-register the acquired brain images with the ARA atlas image. Each individual image was manually matched to its closest corresponding ARA atlas level and the information was manually inserted into a registration table. Although manual, this reduces the 2D registration variability and improves registration accuracy versus using a fully automated registration algorithm purely based on fiducial markers like the Allen Brain Atlas. A 2D diffeomorphic demons algorithm (Vercauteren et al., 2009) was used for image registration. Affine registration was chosen to minimize distortion to preserve axonal morphology. The deformation matrix resulting from the registration process was applied on the original resolution images to attain high-resolution warped images. The algorithm was implemented in Matlab and standalone binaries were generated using Matlab compiler. The Laboratory of Neuro Imaging (LONI) pipeline was used for parallel execution of registration of multiple cases.

### Conflict of interest statement

The authors declare that the research was conducted in the absence of any commercial or financial relationships that could be construed as a potential conflict of interest.

## Abbreviations

AAA: anterior amygdalar area
ACAd: anterior cingulate area, dorsal part
*aco*: anterior commissure, olfactory limb
AMd: anteromedial thalamic nucleus, dorsal part
AOB: accessory olfactory bulb
AON: anterior olfactory nucleus
AONd: anterior olfactory nucleus, dorsal part
AONe: anterior olfactory nucleus, external part
AONm: anterior olfactory nucleus, medial part
AONpv: anterior olfactory nucleus, posteroventral part
ARA: allen reference atlas
BA: bed nucleus of accessory olfactory tract
BDA: biotinylated dextran amine
BST: bed nuclei of stria terminalis
COAa: cortical amygdalar area, anterior part
COApl: cortical amygdalar area, posterior part, lateral zone
COApm: cortical amygdalar area, posterior part, medial zone
CTb: cholera toxin subunit b
DP: dorsal peduncular area
ENT: entorhinal area
ENTl: entorhinal area, lateral part
ENTm: entorhinal area, medial part
FG: Fluorogold
gr: granule cell
*int*: internal capsule
*lot*: lateral olfactory tract
*lotd*: lateral olfactory tract, dorsal part
MA: magnocellular nucleus
MCP: mouse connectome project
MEAav: medial amygdalar area, anteroventral part
mi: mitral cell
MOB: main olfactory bulb
MOBgl: main olfactory bulb, glomerular layer
MOBgr: main olfactory bulb, granule layer
MOBipl: main olfactory bulb, inner plexiform layer
MOBmi: main olfactory bulb, mitral layer
MOBopl: main olfactory bulb, outer plexiform layer
MOp: primary motor area
NLOT: nucleus of the lateral olfactory tract
NT: NeuroTrace^®^
OSN: olfactory sensory neuron
OT: olfactory tubercle
OTl: olfactory tubercle, lateral part
OTm: olfactory tubercle, medial part
PAA: piriform-amygdalar area
PHAL: *Phaseolus vulgaris-leucoagglutinin*
PIR: piriform area
PIRa: piriform area, anterior part
PIR-OT: piriform-olfactory tubercle junction
PIRp: piriform area, posterior part
py: pyramidal cell
RT: reticular nucleus of the thalamus
SSp: primary somatosensory area
SSs: supplemental somatosensory area
tf: tufted cell
TR: postpiriform transition area
TTd: taenia tecta, dorsal part
TTv: taenia tecta, ventral part
VISC: visceral area
VNO: vomeronasal organ.
